# Hypoxia-Inducible Factor 1α Determines Gastric Cancer Chemosensitivity via Modulation of p53 and NF-κB

**DOI:** 10.1371/journal.pone.0012038

**Published:** 2010-08-10

**Authors:** Nadine Rohwer, Christof Dame, Anja Haugstetter, Bertram Wiedenmann, Katharina Detjen, Clemens A. Schmitt, Thorsten Cramer

**Affiliations:** 1 Medizinische Klinik mit Schwerpunkt Hepatologie und Gastroenterologie, Campus Virchow-Klinikum, Charité - Universitätsmedizin Berlin, Berlin, Germany; 2 Fachbereich Biologie, Chemie, Pharmazie, Freie Universität Berlin, Berlin, Germany; 3 Klinik für Neonatologie, Charité - Universitätsmedizin Berlin, Berlin, Germany; 4 Molekulares Krebsforschungszentrum, Charité - Universitätsmedizin Berlin, Berlin, Germany; 5 Max-Delbrück-Centrum für Molekulare Medizin, Berlin, Germany; The Research Institute for Children at Children's Hospital New Orleans, United States of America

## Abstract

**Background:**

Reduced chemosensitivity of solid cancer cells represents a pivotal obstacle in clinical oncology. Hence, the molecular characterization of pathways regulating chemosensitivity is a central prerequisite to improve cancer therapy. The hypoxia-inducible factor HIF-1α has been linked to chemosensitivity while the underlying molecular mechanisms remain largely elusive. Therefore, we comprehensively analysed HIF-1α's role in determining chemosensitivity focussing on responsible molecular pathways.

**Methodology and Principal Findings:**

RNA interference was applied to inactivate HIF-1α or p53 in the human gastric cancer cell lines AGS and MKN28. The chemotherapeutic agents 5-fluorouracil and cisplatin were used and chemosensitivity was assessed by cell proliferation assays as well as determination of cell cycle distribution and apoptosis. Expression of p53 and p53 target proteins was analyzed by western blot. NF-κB activity was characterized by means of electrophoretic mobility shift assay. Inactivation of HIF-1α in gastric cancer cells resulted in robust elevation of chemosensitivity. Accordingly, HIF-1α-competent cells displayed a significant reduction of chemotherapy-induced senescence and apoptosis. Remarkably, this phenotype was completely absent in *p53* mutant cells while inactivation of p53 *per se* did not affect chemosensitivity. HIF-1α markedly suppressed chemotherapy-induced activation of p53 and p21 as well as the retinoblastoma protein, eventually resulting in cell cycle arrest. Reduced formation of reactive oxygen species in HIF-1α-competent cells was identified as the molecular mechanism of HIF-1α-mediated inhibition of p53. Furthermore, loss of HIF-1α abrogated, in a p53-dependent manner, chemotherapy-induced DNA-binding of NF-κB and expression of anti-apoptotic NF-κB target genes. Accordingly, reconstitution of the NF-κB subunit p65 reversed the increased chemosensitivity of HIF-1α-deficient cells.

**Conclusion and Significance:**

In summary, we identified HIF-1α as a potent regulator of p53 and NF-κB activity under conditions of genotoxic stress. We conclude that *p53* mutations in human tumors hold the potential to confound the efficacy of HIF-1-inhibitors in cancer therapy.

## Introduction

Intrinsic and acquired drug resistance are the primary causes for limited efficacy of chemotherapy in the majority of gastrointestinal malignancies, including gastric cancer [1], [2]. Drug resistance represents a complex and multifactorial phenomenon related to tumor microenvironment, e.g. hypoxia, acidosis and inflammation as well as the neoplastic cell itself [3]. Cellular resistance may be inherent to the specific genetic background of the tumor cell or result from mutations and epigenetic alterations after antiproliferative therapy [4], [5].

The transcription factor hypoxia-inducible factor 1 (HIF-1) constitutes a pivotal regulator of cellular adaptation to hypoxia and has been implicated in drug resistance [6]–[8]. The HIF-1 protein is a heterodimer composed of a constitutively expressed β-subunit (ARNT (aryl hydrocarbon receptor nuclear translocator)) and a hypoxia-inducible α-subunit [9]. Under normoxic conditions, HIF-1α activity can be induced by various growth factors, cytokines, activated oncogenes or loss-of-function mutated tumor suppressor genes [10]. HIF-1α is centrally involved in multiple aspects of tumorigenesis including tumor cell proliferation, angiogenesis, metastasis, as well as the response to chemo- and radiotherapy [11]. HIF-1α is overexpressed in a vast number of solid tumors, and tumoral HIF-1α expression is often associated with poor prognosis [12]–[15]. Furthermore, inhibition of HIF-1α by means of RNA interference or pharmacological compounds has proven antitumoral efficacy in various murine cancer models [16]. A contribution of HIF-1α to chemoresistance of neoplastic cells has been observed in a wide spectrum of solid tumors, including gastric cancer [6]–[8], [17]–[20]. However, the underlying molecular mechanisms as well as the role of HIF-1α for drug resistance under normoxic conditions remain largely elusive [8], [18], [21]. Here, we identify suppression of p53 and promotion of nuclear factor κB (NF-κB) activity as central mechanisms for HIF-1α‘s sensitivity-determining role against 5-fluorouracil (5-FU) and cisplatin in human gastric cancer cells.

## Results

### HIF-1α determines sensitivity of gastric cancer cells towards the chemotherapeutic agents 5-FU and cisplatin

**Figure 1:**
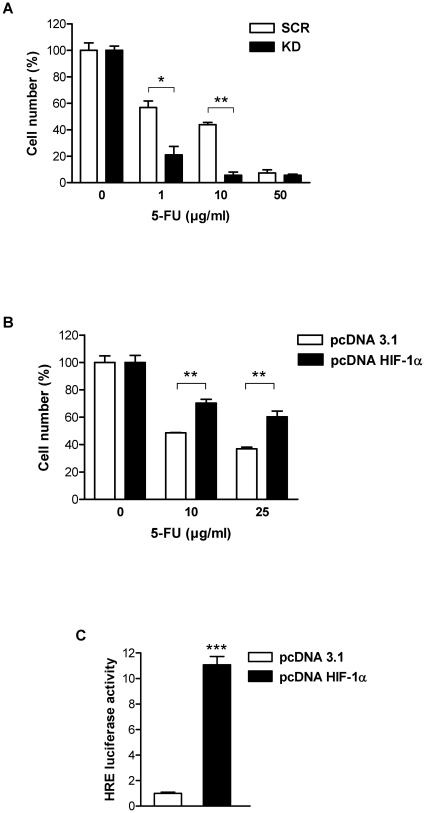
HIF-1α mediates resistance towards the chemotherapeutic agent 5-FU in AGS cells. (A) Proliferation of AGS KD and SCR cells 24 h after treatment with increasing concentrations of 5-FU under normoxic conditions. Cell numbers are shown as percent of untreated cells (*, *P*<0.05; **, *P*<0.01). (B) AGS wild-type cells were transfected with HIF-1α expression vector (pcDNA HIF-1α) or empty vector (pcDNA 3.1) and treated with 5-FU 24 h post transfection. Cell numbers were determined 24 h after treatment with 5-FU, and are shown as percent of untreated control cells (**, *P*<0.01). (C) AGS wild-type cells were co-transfected with either pcDNA HIF-1α or pcDNA 3.1 plus HRE-Luciferase reporter (pHRE-Luc) and *Renilla* reporter (phRL-null) as internal control. Cells were harvested 48 h post transfection. HRE luciferase activity, normalised for *Renilla* luciferase activity, was expressed relative to that of control transfected cells (***, *P*<0.001).

Functional inactivation of HIF-1α was achieved by lentiviral transduction of AGS and MKN28 cells with small interfering RNA (siRNA) specifically targeting HIF-1α. This experimental approach yielded a highly efficient knockdown demonstrated by a near complete failure of transduced cells to induce HIF-1α protein in response to hypoxia as published previously [22]. To evaluate the importance of HIF-1α for the sensitivity of human gastric cancer cells towards established chemotherapeutic agents, we compared the effects of 5-FU and cisplatin in HIF-1α-competent (scrambled, “SCR”) and HIF-1α-deficient (knockdown, “KD”) AGS cells. Functional inactivation of HIF-1α shifted the dose dependency of growth inhibition towards lower drug concentrations (Figure 1A and Figure S1), suggesting that HIF-1α is capable to reduce chemotherapy susceptibility of gastric cancer cells under normoxic conditions. In line with previous reports [6]–[8], [17], [18], exposure to hypoxia increased resistance to 5-FU in AGS cells, however inactivation of HIF-1α resulted in robust elevation of chemosensitivity under hypoxic conditions (Figure S2). In a complementary approach, we studied the consequences of overexpressing HIF-1α (pcDNA HIF-1α) for the chemosensitivity of AGS cells. AGS cells overexpressing HIF-1α were considerably more resistant to treatment with 5-FU (Figure 1B). Stable HIF-1α expression was confirmed by HRE (hypoxia responsive element) luciferase reporter assay (Figure 1C). These results strongly suggest that HIF-1α limits the cytotoxic action of 5-FU and cisplatin in human gastric cancer cells and that inactivation of HIF-1α may have beneficial effects on chemosensitivity.

### HIF-1α limits chemotherapy-induced cell cycle arrest and apoptosis via suppression of p53

**Figure 2:**
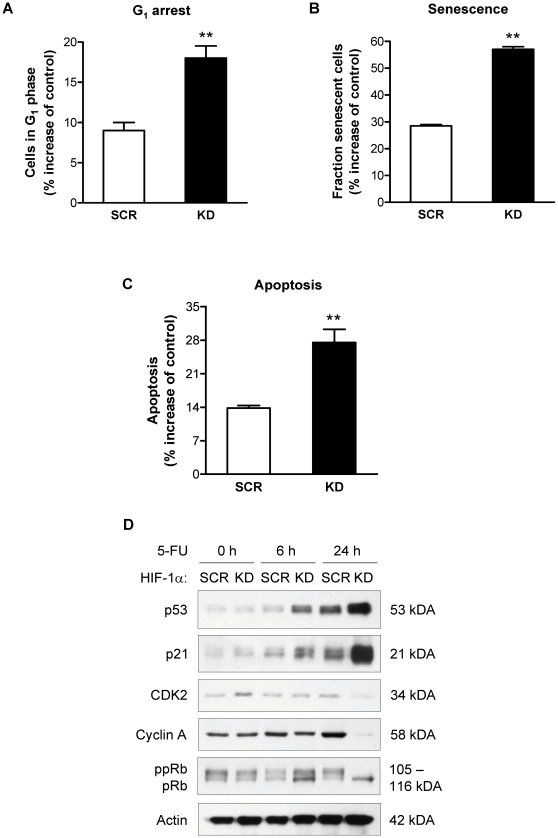
HIF-1α mediates resistance towards 5-FU by blocking p53-dependent G_1_ arrest and apoptosis. (A) AGS KD and SCR cells were treated for 24 h with 10 µg/ml 5-FU, and cell cycle distribution was determined by FACS analysis (**, *P*<0.01). (B) Chemotherapy-induced senescence was quantified in AGS KD and SCR cells 4 days after treatment with 5-FU by measurement of SA-β-Gal activity (**, *P*<0.01). (C) AGS KD and SCR cells were treated for 48 h with 10 µg/ml 5-FU and apoptosis was quantitated based on detection of active caspase-3 using flow cytometry (**, *P*<0.01). (D) Representative immunoblot analysis of p53, p21, CDK2, cyclin A and pRb protein levels in AGS KD and SCR cells treated with 10 µg/ml 5-FU for 6 and 24 h. Actin served as loading control. ppRb, phosphorylated pRb.

We started a characterization of the observed growth inhibition by analyzing cell cycle distribution patterns after chemotherapy. G_1_-synchronized, serum-starved AGS cells were released from G_0_/G_1_ phase by addition of serum and cell cycle profiles were determined following the addition of 5-FU. Released cultures of untreated AGS readily progressed through G_1_ into S and G_2_/M phases [22], whereas 5-FU-treated cells remained in G_1_ phase (not shown). Interestingly, the 5-FU-dependent retention of cells in G_1_ phase was greatly augmented in AGS KD compared to AGS SCR cells, consistent with G_1_ cell cycle arrest (Figure 2A). Irreversible cell cycle arrest has emerged as an important mode of action of antiproliferative agents and is characterized by cellular features of senescence [7], [23]. Accordingly, the fraction of senescent cells was determined. Indeed, treatment with 5-FU led to a robust induction of senescence in AGS cells. This response was significantly enhanced in cells with concurrent loss of HIF-1α (Figure 2B). Furthermore, induction of apoptosis was suggested by an increased pre G_1_ fraction in DNA histograms of 5-FU-treated AGS KD cells (not shown). Therefore, a quantitative analysis of the apoptotic cell fraction was obtained based on the detection of cleaved caspase-3 (Figure 2C). Consistent with the data on cell cycle distribution, the 5-FU-induced apoptotic fraction was significantly increased in HIF-1α-deficient AGS KD cells as compared to HIF-1α-competent cells.

Chemotherapy-induced senescence and apoptosis both have been intimately linked to the tumor suppressor *p53*. Thus, we hypothesized that p53 might contribute to augmented cytotoxicity of 5-FU upon loss of HIF-1α. After 5-FU treatment, p53 protein gradually accumulated in AGS cells, an effect that was strikingly enhanced in HIF-1α-deficient AGS cells (Figure 2D). This stabilization of p53 was associated with increased levels of the cyclin-dependent kinase (CDK) inhibitor p21, a well established transcriptional target and downstream effector of p53 with functions in cell cycle arrest, senescence induction and apoptosis (Figure 2D). Again, HIF-1α-deficient AGS cells showed a markedly stronger increase in p21 than HIF-1α-proficient AGS cells. Strong induction of p21 is expected to inhibit the activity of G_1_ cyclin/CDK complexes, resulting in hypophosphorylation of retinoblastoma protein (pRb) and failure to induce S phase cyclins, e.g. cyclin A. Indeed, both pRb hypophosphorylation and reduced cyclin A levels were confirmed in 5-FU-treated AGS KD cells and - to a lesser extent - also in AGS SCR cells (Figure 2D). These changes corroborate the G_1_ phase retention observed in DNA histograms and are consistent with the irreversible G_1_ arrest observed in chemotherapy-induced senescence. Thus, the different biological outcomes of 5-FU treatment in HIF-1α-deficient and –proficient AGS cells arise from differential regulation of p53 and its downstream target p21.

### Inactivation of p53 blunts the role of HIF-1α for chemosensitivity

**Figure 3:**
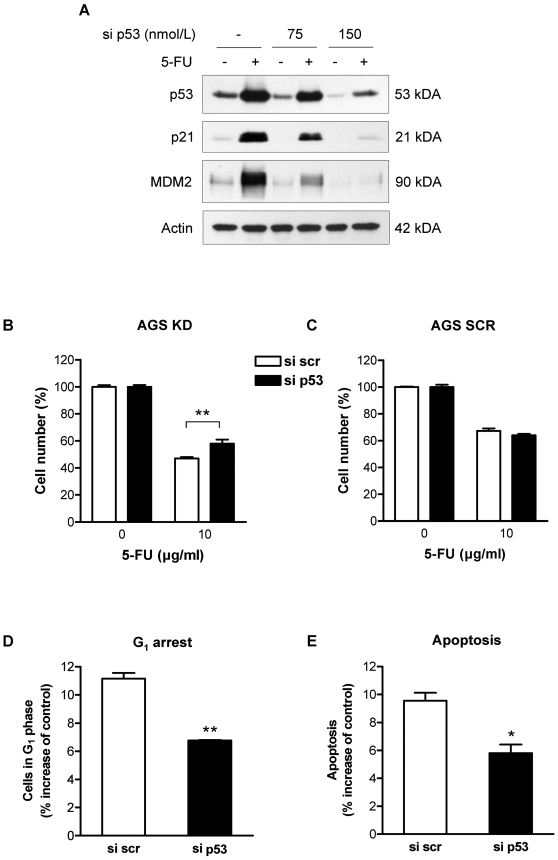
siRNA silencing of p53 reverses the chemosensitization of AGS KD cells. AGS cells were transfected with control siRNA (si scr) or siRNA against p53 (si p53), and 10 µg/ml 5-FU was added 24 h post transfection. (A) Immunoblot analysis for p53, p21 and MDM2 in whole cell extracts from AGS KD cells after 5-FU treatment. Actin served as loading control. (B and C) Cell numbers of si scr and si p53 transfected AGS cells was determined 24 h after treatment with 5-FU. Results are shown as percent of untreated control cells (**, *P*<0.01). Cells in the G_1_ phase (D) and thesub-G_1_ population (E) were evaluated from DNA histograms of AGS KD cells transfected with si p53 or si scr and treated for 24 h with 5-FU (*, *P*<0.05; **, *P*<0.01).

To obtain experimental evidence for the proposed role of p53 in HIF-1α-mediated regulation of chemosensitivity in AGS cells, we functionally inactivated p53 by RNA interference using transient transfection of anti-p53 siRNA (si p53), or a scrambled control siRNA (si scr). P53 was efficiently knocked down, as indicated by the failure of the transfected cells to induce the p53 effectors p21 and MDM2 in response to 5-FU treatment (Figure 3A). Interestingly, AGS KD cells transfected with si p53 were significantly less susceptible to growth inhibition by 5-FU than AGS KD cells transfected with control siRNA (Figure 3B). In line with these findings, G_1_ cell cycle retention and apoptosis of 5-FU-treated AGS KD cells were reduced by p53 knockdown in comparison to cells transfected with control siRNA (Figure 3D and 3E). In sharp contrast, chemosensitivity of HIF-1α-proficient AGS cells was not influenced by inactivation of p53 (Figure 3C).

### HIF-1α fails to affect chemosensitivity in *p53* mutant cells

**Figure 4:**
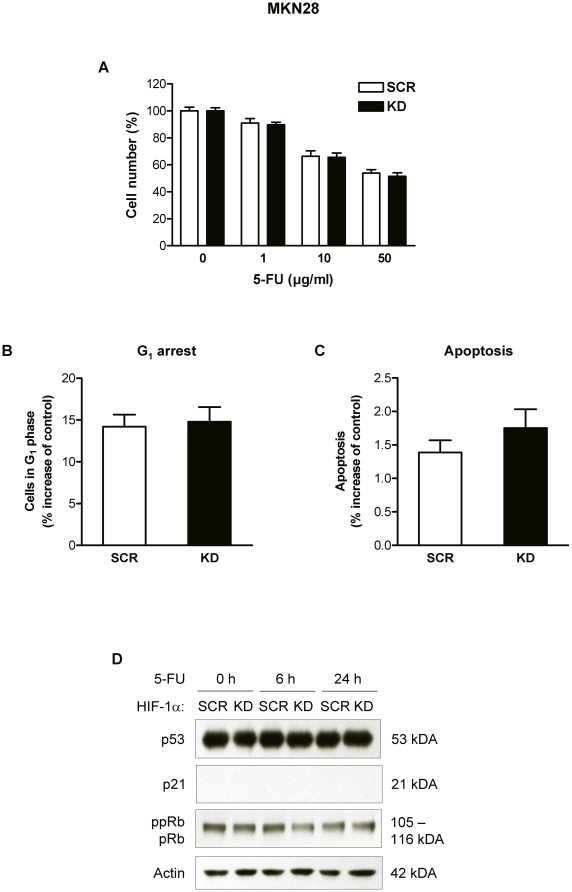
Effects of 5-FU on MKN28 cells with mutant *p53.* (A) Cell numbers of MKN28 KD and SCR cells 24 h after treatment with 5-FU under normoxic conditions. Data are shown as percent of untreated cells. Cells in G_1_ phase (B) and apoptotic cells (C) were quantitated from DNA histograms of MKN28 KD and SCR cells treated for 48 h with 10 µg/ml 5-FU. (D) Immunoblot analysis of p53, p21 and pRb protein levels in MKN28 KD and SCR cells treated with 10 µg/ml 5-FU for 6 and 24 h. Actin served as loading control.

To confirm the HIF-1α-dependent regulation of 5-FU responsiveness and to further characterize the contribution of p53, we examined a second human gastric cancer cell line (MKN28), which carries a missense mutation in *TP53* at codon 251. Interestingly, deletion of HIF-1α in MKN28 cells failed to enhance the growth inhibition after exposure to 5-FU (Figure 4A). Similarly, 5-FU-induced G_1_ accumulation and apoptosis of MKN28 cells were not affected by loss of HIF-1α (Figure 4B and 4C). In line with these findings, the protein levels of p53 and pRb remained unchanged in 5-FU-treated MKN28 cells throughout the 24 h period, and p21 induction was absent (Figure 4D). However, when p53 function was restored by pretreatment with the chemical compound PRIMA-1 [24] HIF-1α knockdown translated into a significantly enhanced 5-FU cytotoxicity (Figure S3). Consistent with the established role of p53 in chemotherapy-induced cytotoxic/cytostatic effects, treatment with PRIMA-1 *per se* slightly reduced the proliferation of MKN28 cells and significantly enhanced the efficacy of 5-FU in MKN28 cells (Figure S3).

### NF-κB is an important mediator of HIF-1α’s role in chemosensitivity

**Figure 5:**
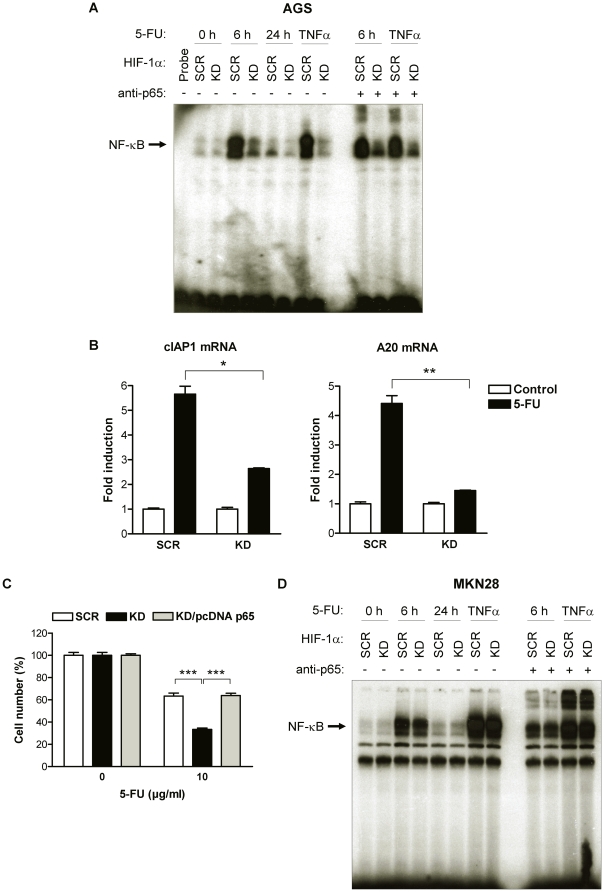
HIF-1α-mediated activation of NF-κB limits the toxicity of 5-FU. (A) Nuclear extracts of AGS KD and SCR cells were prepared at the indicated time points after treatment with 10 µg/ml 5-FU or TNFα as a positive control, and DNA-binding activity for NF-κB was examined by EMSA. For supershift experiments, nuclear extracts were incubated with an anti-p65 antibody. (B) Expression of NF-κB target genes cIAP1 and A20 mRNA in total RNA extracts from AGS KD and AGS SCR cells 48 h after treatment with 10 µg/ml 5-FU. Data were expressed relative to mRNA levels in untreated AGS SCR cells, set at 1.0 (*, *P*<0.05; **, *P*<0.01). (C) AGS KD cells were co-transfected with either pcDNA p65 or pcDNA 3.1 and treated with 5-FU 24 h post transfection. Cell numbers were after another 24 h and are presented as percent of untreated control cells (***, *P*<0.001). (D) DNA binding activity for NF-κB was examined by EMSA using nuclear extracts of MKN28 KD and SCR cells treated with 10 µg/ml 5-FU or TNFα for the indicated times. Antibody inhibition was performed with an anti-p65 antibody.

Activation of NF-κB is associated with protection from chemotherapy-induced apoptosis and, conversely, inhibition of NF-κB can enhance the efficacy of anti-neoplastic agents both *in vivo* and *in vitro* [25]–[27]. Therefore, we determined NF-κB DNA-binding activity in HIF-1α-deficient and –proficient AGS cells after treatment with 5-FU by electrophoretic mobility shift assay (EMSA). Treatment with 5-FU potently activated NF-κB DNA-binding in AGS SCR cells, with peak levels occurring 6 h after exposure to 5-FU (Figure 5A). Treatment with TNFα for 4 h served as positive control for activation of NF-κB. Furthermore, a supershift was induced by an anti-p65 antibody, confirming that 5-FU induced NF-κB complexes contained the 65-kDa subunit (p65). Loss of HIF-1α significantly inhibited activation of NF-κB in response to 5-FU and TNFα (Figure 5A). Consistent with this observation, 5-FU treatment also failed to induce the NF-κB target genes cIAP1 and A20 in AGS KD cells, whereas they were readily induced in AGS SCR cells (Figure 5B).

To address the functional significance of NF-κB for 5-FU-induced growth inhibition, we overexpressed p65 (pcDNA p65) in AGS KD cells. Transfection of pcDNA p65, but not the empty control vector, resulted in a significant induction of p65 protein and NF-κB transcriptional activity in AGS KD cells (Figure S4). Of note, HIF-1α-deficient AGS KD cells overexpressing p65 were considerable more resistant to 5-FU treatment compared to AGS KD cells transfected with the control vector (Figure 5C), consistent with an essential role of NF-κB in mediating chemoresistance towards 5-FU in gastric cancer cells. Taken together, a concurrent activation of p53 and inhibition of NF-κB in 5-FU-treated, HIF-1α-deficient AGS cells was observed. To clarify, whether both events are interdependent, we studied 5-FU-induced NF-κB activation in MKN28 cells with mutant *p53.* Both 5-FU and TNFα activated NF-κB DNA-binding in a time-dependent manner, indicating p53-independent mechanisms of NF-κB activation by 5-FU (Figure 5D). However, different from the finding in AGS cells, this NF-κB activation in the *p53* mutant cell line was not blunted by HIF-1α inactivation. Thus, HIF-1α may support chemotherapy-induced NF-κB activation by counteracting p53-dependent inhibitory mechanisms.

### Altered ROS formation is responsible for HIF-1α-induced modification of p53 activity

**Figure 6:**
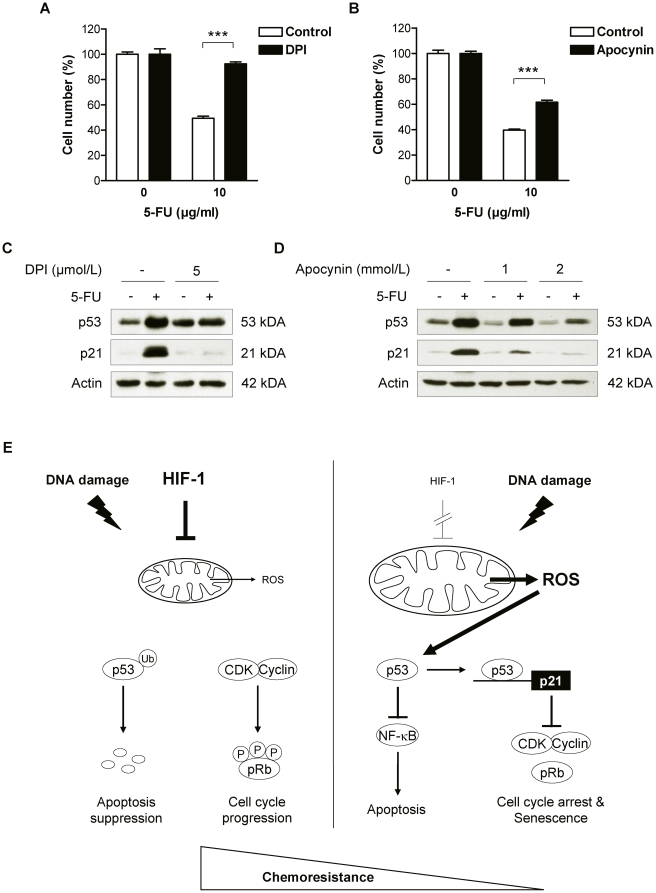
ROS as molecular mediator of the HIF-1α effect on chemosensitivity in AGS KD cells. (A–D) AGS KD cells were pretreated for 16 h with different concentrations of the NADPH oxidase inhibitors diphenyleneiodonium (DPI) or apocynin. Proliferation of DPI-pretreated (A) or apocynin-pretreated (B) AGS KD cells 24 h after treatment with 10 µg/ml 5-FU under normoxic conditions (***, *P*<0.001). Cell numbers are shown as percent of untreated cells. (C and D) The effect of DPI (C) and apocynin (D) on p53 and p21 protein levels was determined by immunoblot analysis using whole cell extracts of AGS KD cells treated for 24 h with 10 µg/ml 5-FU. (E) Proposed model for HIF-1-dependent regulation of chemosensitivity by ROS-induced modulation of p53. (left panel) Chemotherapy-induced response in HIF-1-competent cells. HIF-1 counteracts generation of ROS at the mitochondrial level. Decreased ROS levels in turn abate activation of p53 and allow for cell cycle progression despite chemotherapy. Hence, HIF-1-competent cells display a more chemoresistant phenotype. Ub, ubiquitin; P, phosphate. (right panel) Chemotherapy-induced response in HIF-1-deficient cells. Inactivation of HIF-1 leads to accelerated mitochondrial ROS generation. ROS are potent inducers of p53 and thus boost activation of p53 by chemotherapeutic agents. P53 in turn transactivates -among others - the cyclin-dependent kinase (CDK) inhibitor p21 and inhibits NF-κB activity. The combined activation of p53 and inhibition of NF-κB, result in apoptosis and/or senescence of HIF-1-deficient cells, hence a more chemosensitive phenotype.

To clarify the molecular mechanism underlying p53 superinduction in 5-FU-treated HIF-1α-deficient cells, we characterized the role of reactive oxygen species (ROS). ROS constitute a candidate link as (i) ROS are potent activators of p53 function and considered key factors in the induction of p53 by various chemotherapeutic agents [28], and (ii) HIF-1α can suppress ROS generation by decreasing mitochondrial activity and biogenesis [22], [29], [30]. Accordingly, AGS KD cells were pretreated with the ROS-inhibitors diphenyleneiodonium chloride (DPI) or apocynin. Both inhibitors conferred significant protection against 5-FU-induced growth inhibition in AGS KD cells (Figure 6A and 6B). Furthermore, DPI and apocynin almost completely prevented the induction of p53 and its downstream target p21 in 5-FU-treated cells (Figure 6C and 6D). These results suggest an intersection of HIF-1α signalling with the p53-mediated response to 5-FU at the level of ROS production. To establish a causal role of HIF-1α for the redox potential of AGS cells after 5-FU treatment, intracellular ROS levels were determined in AGS KD and SCR cells by flow cytometry. We found that the intracellular superoxide levels in 5-FU-treated AGS KD cells were 2.5-fold higher than those in 5-FU-treated AGS SCR cells (Figure S5), indicating that functional inactivation of HIF-1α in AGS cells resulted in significant and functional elevation of intracellular oxidative stress even under chemotherapeutic treatment.

## Discussion

The transcription factor HIF-1α has been established as important mediator of hypoxia-mediated chemoresistance [6]–[8], [17], [18], [20]. Here, we identify HIF-1α as a powerful determinant of chemosensitivity in gastric cancer cells under normoxic conditions. By applying a lentivirus-based siRNA system we show significantly enhanced 5-FU and cisplatin toxicity in HIF-1α-deficient gastric cancer cells. Available data on the role of HIF-1α for the chemosensitivity of cancer cells under normoxic conditions are conflicting. While HIF-1α-deficient fibrosarcoma cells (HT1080) displayed significantly enhanced sensitivity towards etoposide under ambient air, colon cancer (HCT116) and hepatoma (Hepa-1) cells failed to do so [6]. Unruh *et al.* reported enhanced susceptibility of HIF-1α-deficient murine embryonic fibroblasts to carboplatin or etoposide under normoxic as well as hypoxic conditions [8]. With respect to gastric cancer, enhanced efficacy of 5-FU and vincristine was demonstrated under normoxia *in vitro* [18]. Well in line with our results, both studies concluded a pivotal role for HIF-1α in mediating chemoresistance under normoxic conditions. Interestingly, a recent study from Japan demonstrated lower efficacy of 5-FU-based chemotherapy in HIF-1α-expressing human gastric adenocarcinomas, strengthening the perception of HIF-1 as a pivotal factor in the determination of gastric cancer chemoresistance [31].

Control of cancer progression by chemotherapy relies at least in part on induction of cellular senescence. Recently, loss of HIF-1α was shown to cause premature senescence of immortalized murine embryonic fibroblasts under normoxic conditions [32]. Our current data suggests that HIF-1α similarly guards gastric cancer cells against chemotherapy-induced senescence under normoxic conditions. This constitutes the first report of elevated chemotherapy-induced senescence via functional inactivation of HIF-1α in an established human cancer cell line. In HIF-1α-deficient cells, we also observed improved apoptosis induction in response to 5-FU. Previous studies reported a reactivation of the proapoptotic factor Bid [6], or a change in the balance of pro- and antiapoptotic Bcl-2 family members to account for increased apoptosis rates following inactivation of HIF-1α in drug-treated gastric cancer cells [18].

Our current study identifies a novel mechanism, whereby HIF-1α counteracts both chemotherapy-induced senescence and apoptosis: We present conclusive evidence for the capacity of HIF-1α to suppress the induction of the tumor suppressor p53 in response to 5-FU under normoxic conditions. P53 is a pivotal cell fate determinant due to its role in regulating cell-cycle progression and apoptosis in response to cellular stress and constitutes the most commonly mutated gene in human cancers [33]. A wide variety of chemotherapeutic agents were shown to stabilize p53 and, conversely, loss of p53 constitutes a principle mechanism of cancer resistance towards chemotherapy [33], [34]. The interaction of p53 and HIF-1α has been the subject of longstanding debates as both positive and negative reports have been published [35]. However, the entire previously published work focussed on p53-HIF-1α-interactions under hypoxic (or even anoxic) conditions. To the best of our knowledge, our experiments for the first time provide evidence for the suppression of p53 activity by HIF-1α under normoxic conditions. As consequence of p53 upregulation in HIF-1α-deficient cells, we observed changes in downstream effectors that are linked to the irreversible cell cycle arrest characteristic of senescence, e.g. p21 stabilization and hypophosphorylation of pRb. Different from our observation, recent work on chemoresistance towards etoposide in HIF-1α-deficient immortalized murine embryonic fibroblasts did not observe an induction of p21 [36]. Also, HIF-1α stabilized p21 and p27 as well as led to hypophosphorylation of pRb during hypoxia-induced growth arrest of immortalized murine embryonic fibroblasts and primary splenic B-lymphocytes [37]. These contrasting results are most likely explained by the investigated cell types: While Goda *et al*. characterized a physiological response to hypoxia in non-transformed cell types, we analyzed the response to severe DNA damage in established cancer cell lines.

While p53 was repeatedly shown to counteract NF-κB function [38], [39], our current data indicate a role for the tumor suppressor in the regulation of HIF-1α-dependent NF-κB activation. Apart from p53, NF-κB has emerged as a second, central determinant of resistance towards chemotherapeutic agents [40]. Several different studies have established functional links between NF-κB and HIF-1α, though they variably place HIF-1α either upstream of NF-κB or vice versa. For instance, hypoxia-induced stabilization of HIF-1α in smooth muscle cells is under transcriptional control of NF-κB [41]. Similarly, results obtained from conditional IKK-β knockout mice confirmed the pivotal role of NF-κB in controlling basal and hypoxia-induced expression of HIF-1α *in vivo* [42]. Conversely, gene expression of the NF-κB subunit p65 was demonstrated to be controlled by HIF-1α in the context of hypoxia-suppressed apoptosis of neutrophils [43]. Our finding of markedly reduced NF-κB activity in HIF-1α-deficient cells upon treatment with 5-FU therefore is well in line with this latter report. Interestingly, we also observed significantly reduced DNA binding of NF-κB subunits in HIF-1α-deficient cells after stimulation with TNFα, a well established inducer of NF-κB activity [44]. This raises the pertinent question under which physiological and pathophysiological conditions HIF-1α is able to regulate NF-κB activation. HIF-1α and NF-κB share crucial importance for various processes such as inflammation, microbial killing and tumorigenesis. The exact molecular nature as well as the hierarchy of their interaction is most likely cell- and context-dependent and can not be generalized.

In the current study we were able to pinpoint ROS as an intersection point of HIF-1α with the p53-mediated cellular stress response to chemotherapy. Intracellular ROS are known as potent inducers of p53 and participate in the activation of p53 by chemotherapeutic drugs [45]. Mitochondria represent the prime source of intracellular ROS [46], and HIF-1α likely counteracts ROS production at the mitochondrial level via multiple mechanisms including inhibition of mitochondrial biogenesis and of pyruvate shuttling into the mitochondria, reduction of mitochondrial activity due to enhanced utilization of glycolysis and activation of mitochondrial autophagy [29], [30], [47], [48]. Previously, we established a functional link between HIF-1α-controlled reduction of ROS and anchorage independence of gastric cancer cells [22], implicating HIF-1α in the pathogenesis of gastric cancer in the absence of hypoxia. We now find that the capacitiy of HIF-1α to restrict ROS production of gastric cancer cells also confers resistance to chemotherapeutic agents that function via activation of p53 (Figure 6E). Interestingly, increasing effects on therapy resistance via modulation of p53 and ROS have also been reported for HIF-2α [49]. The HIF-α isoforms 1 and 2 show a wide overlap in putative HIF targets and binding to hypoxic response elements and definite allocation of hypoxia-induced effects to either isoform is not always accomplishable [50]. Bertout *et al.* demonstrated that inhibiting HIF-2α increases radiation-induced apoptosis via ROS accumulation and subsequent augmentation of p53 activity [49]. In addition, Roberts *et al.* showed that resistance against chemotherapy is partially mediated by HIF-2α-mediated suppression of p53 in renal cell carcinoma cells [51]. Hence, the herewith reported observations warrant investigations into the potential role of HIF-2α, a task that is currently under way in our laboratory.

In view of the clinical need to identify response predictors for available treatment options, our results could potentially direct treatment decisions: on one hand, knowledge of HIF-1α overexpression could direct a choice of drugs that largely act in a p53-independent fashion. On the other hand, a particular benefit may result from combining HIF-1-inhibitors and DNA damaging agents (e.g. 5-FU) in cancers with functional p53. Conversely, a reduced efficacy of HIF-1-inhibitors might be anticipated for treatment of p53 defective tumors, an aspect that may constitute a confounding factor in clinical trials of HIF-1α-inhibiting treatment regimes.

## Materials and Methods

### Cell culture and chemicals

AGS (CRL-1739, ATCC, Rockville, Maryland, USA) and MKN28 (JCRB Cell Bank, Tokyo, Japan) cells were grown as monolayer cultures in standard medium. Generation of AGS and MKN28 cells stably expressing either siRNA specifically targeting HIF-1α (knockdown, “KD”) or unspecific control siRNA (scrambled, “SCR”) was published previously [22]. 5-fluorouracil (5-FU), cis-Diammineplatinum(II) dichloride (cisplatin) and the superoxide anion inhibitors diphenyleneiodonium chloride (DPI) and apocynin were purchased from Sigma-Aldrich (Germany) and dissolved in DMSO. PRIMA-1 (for p53-reactivation and induction of massive apoptosis) was obtained from Tocris Biosciences (Ellisville, Missouri, USA) and dissolved in sterile water. Vehicle control of the solvents was included in all experiments.

### Cell proliferation assay

For determination of cell growth, 3×10^4^ cells were seeded in triplicate into 24-well plates, allowed to attach for 16 h and then treated as indicated under normoxic or hypoxic conditions. After treatment, cells were trypsinized, and viable cells were counted using a hemocytometer.

### Determination of cell cycle distribution and apoptosis by flow cytometry

Cell cycle distribution including the pre-G_1_ fraction was determined from DNA histograms as described [52]. Apoptosis was also quantitated from detection of active, cleaved caspase-3 by flow cytometry using an Alexa Fluor® 488-conjugated antibody (Cell Signaling Technology, Danvers, Massachusetts, USA).

### Quantification of senescence

Senescence-associated β-galactosidase activity was assessed in cytospin preparations as described [53].

### Immunoblot analysis

Whole cell lysates were prepared as previously described [52], then resolved on a 10% sodium dodecyl sulfate-polyacrylamide gel and transferred to nitrocellulose (Amersham Biosciences, Freiburg, Germany). Blots were probed with antibodies against p53 and CDK2 (Santa Cruz Biotechnology, Santa Cruz, California, USA), p21 (Oncogene Research Products, Bad Soden, Germany), cyclin A (Upstate, Temecula, California, USA), pRb (BD Pharmingen, Heidelberg, Germany), MDM2 (Calbiochem, San Diego, California, USA), p65 (Cell Signaling Technology) and actin (Sigma-Aldrich). Secondary antibodies were conjugated to Horseradish Peroxidase (Dianova, Hamburg, Germany) and peroxidase activity was visualized using the Western Lightning Chemiluminescence Reagent Plus (Perkin Elmer Life Sciences, Boston, Massachusetts, USA).

### Quantitative real-time PCR analysis

For real-time PCR analysis, total cellular RNA was extracted with Trizol reagent (Invitrogen, Karlsruhe, Germany). First strand cDNA was synthesized with an oligo (dT) primer and a SuperScript™ First Strand Synthesis System (Invitrogen). Quantitative real-time PCR analysis was performed by using TaqMan PCR Universal Mastermix (for β-actin) or SYBR GREEN PCR Master Mix (for A20 and cIAP1; Applied Biosystems, Darmstadt, Germany). Primer sequences are supplied in Table S1. To normalize the amount of input RNA, PCR reactions were done with probe and primers for β-actin.

### Transient transfection and reporter luciferase assay

Transient transfections of AGS cells were carried out using Effectene Transfection Reagent (Qiagen, Hilden, Germany) according to the manufacturer's protocol. For overexpression studies, cells were seeded at 3×10^4^ cells/24-well and transfected with 100 ng of pcDNA HIF-1α (kindly provided by Wanja Bernhardt, Universitätsklinikum Erlangen, Erlangen, Germany) or pcDNA p65 (kindly provided by Hiroyasu Nakano, Jutendo University, Tokyo, Japan), respectively. For HRE or NF-κB luciferase assay, 3×10^4^ cells/24-well were co-transfected with 100 ng of pHRE-Luc (a gift from Randall S. Johnson, UCSD, San Diego, California, USA) or IgκB-Luc (a gift from Florian R. Greten, Technische Universität München, München, Germany), and 30 ng of phRL-null (Promega, Mannheim, Germany). Luciferase activity was measured with the Dual Luciferase Reporter Assay System (Promega) as described [54]. To achieve transient p53 suppression, AGS cells were transfected at 30% confluence with 75 or 150 nmol/L si p53, (*Silencer* Select siRNA, Applied Biosystems) and analyzed 48 h after transfection. A non-specific siRNA (si scr, Eurogentec, Seraing, Belgium) was used as control.

### Electrophoretic mobility shift assay (EMSA)

Nuclear protein extracts were prepared as described [54]. EMSA was performed as previously described [55] using 8 µg of nuclear protein and 100 fmol/L of the end-radiolabeled 22 bp double stranded NF-κB consensus oligonucelotide (forward strand: 5′-AGT TGA GGG GAC TTT CCC AGG C-3′; E3292, Promega). Samples were resolved by electrophoresis on a non-denaturating 5% polyacrylamide gel. After drying of the gel, complex formation was visualized by autoradiography. For supershift experiments, an anti-p65 antibody was added (Santa Cruz Biotechnology) prior to the radiolabeled probes.

### Measurement of intracellular superoxide levels

Intracellular superoxide anion levels were estimated using the fluorescent dye dihydroethidium (DHE), obtained from Sigma-Aldrich. After 24 h cultivation, cells were trypsinized, harvested by centrifugation, resuspended in PBS containing 10 µmol/L DHE for 251 min at 37°C and thereafter washed with ice-cold PBS. Dye oxidation was determined by flow cytometry with excitation and emission settings of 488 and 585 nm, respectively. The mean fluorescence intensity of at least 1×10^5^ cells was analyzed and corrected for autofluorescence from unlabeled cells.

### Statistical analysis

Shown are means ± SEM of at least three independent experiments. Statistical analysis was performed by two-tailed Student *t* test using Prism 4.0 software (GraphPad Software, San Diego, California, USA). Differences were considered statistically significant at *P*<0.05.

## Supporting Information

Figure S1 HIF-1α mediates resistance towards the chemotherapeutic agent cisplatin in AGS cells. Proliferation of AGS KD and SCR cells 48 h after treatment with increasing concentrations of cisplatin under normoxic conditions (**, P<0.01). Values represent the mean ± SEM of triplicate determinations and cell numbers are shown as percent of untreated cells.
(1.81 MB TIF)

Figure S2 HIF-1α mediates resistance towards the chemotherapeutic agent 5-FU under hypoxic conditions. Proliferation of AGS KD and SCR cells 24 h after treatment with increasing concentrations of 5-FU under hypoxic conditions (**, P<0.01; ***, P<0.001). Values represent the mean ± SEM of triplicate determinations and cell numbers are shown as percent of untreated cells.
(1.81 MB TIF)

Figure S3 Restoration of wild-type p53 by PRIMA-1 in MKN28 cells with mutant p53. MKN28 KD and SCR cells were pretreated with 30 µmol/L PRIMA-1 for 6 h and then exposed to 10 µg/ml 5-FU. Cell growth was determined 24 h after treatment with 5-FU (**, P<0.01). Values represent the mean ± SEM of triplicate determinations and cell numbers are shown as percent of untreated cells.
(1.81 MB TIF)

Figure S4 Overexpression of NF-κB subunit p65 in AGS KD cells. AGS KD cells were co-transfected with either pcDNA p65 or pcDNA 3.1 plus IgκB-Luc and phRL-null as internal control. Cells were harvested 48 h post transfection and NF-κB luciferase activity, normalised for Renilla luciferase activity, was expressed relative to control transfected cells (***, P<0.001). Bottom panel shows immunoblot analysis of p65 and actin 48 h post transfection.
(1.72 MB TIF)

Figure S5 Analysis of superoxide anion levels in AGS cells after 5-FU treatment. AGS KD and SCR cells were treated for 24 h with 10 µg/ml 5-FU under normoxia, stained with 10 µmol/L dihydroethidium, and dye oxidation was determined by flow cytometry. Values represent the mean ± SEM of three independent experiments (**, P<0.01).
(1.77 MB TIF)

Table S1 List of primer and probe sequences.
(1.77 MB TIF)
