# Supplementary figures and images for: Hypoxia-Inducible Factor 1α Determines Gastric Cancer Chemosensitivity via Modulation of p53 and NF-κB
Source: PLoS One. 2010 Aug 10;5(8):e12038. doi: 10.1371/journal.pone.0012038 (PMC2919384; doi:10.1371/journal.pone.0012038)

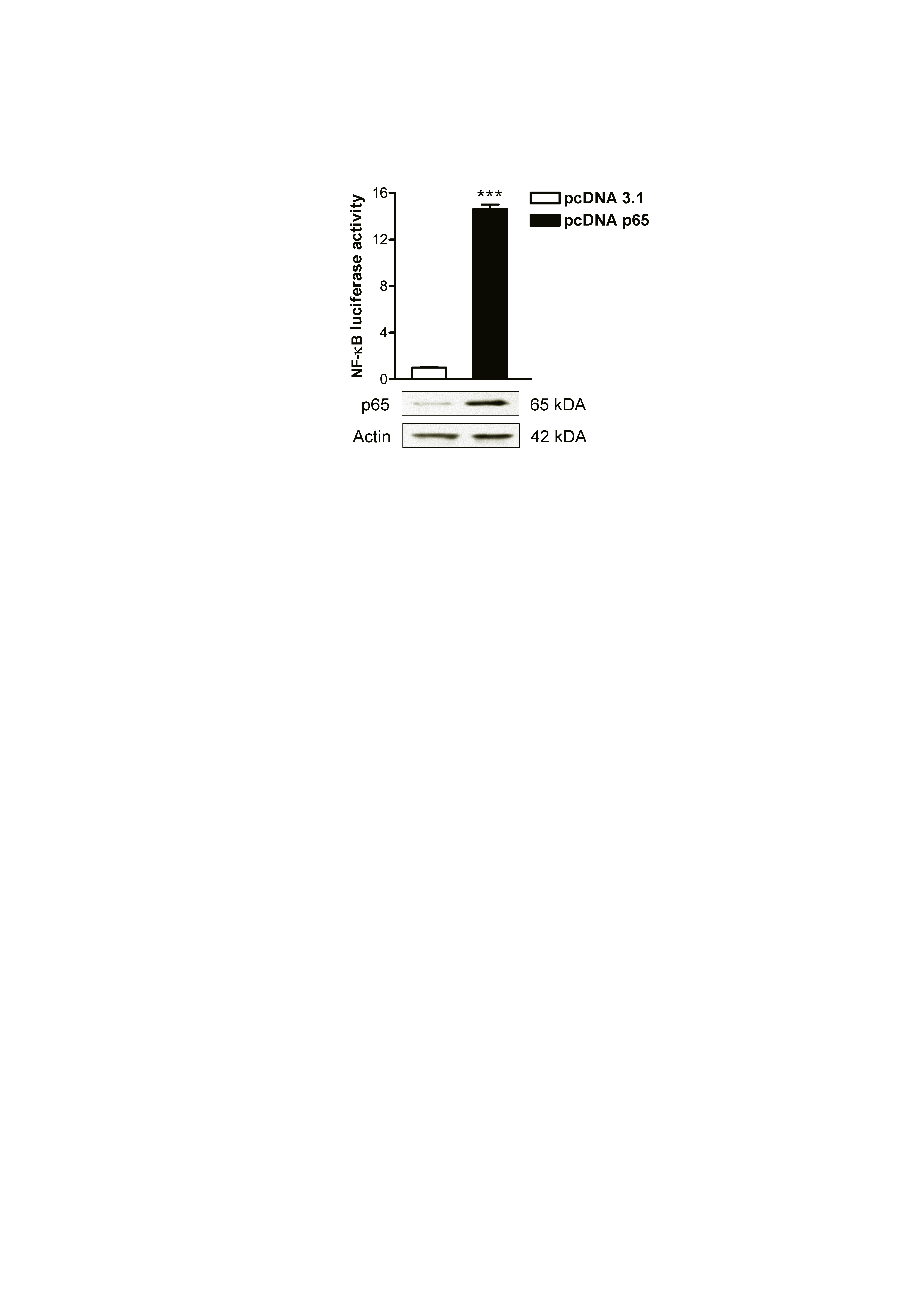

Supplement: Figure S4 — Overexpression of NF-κB subunit p65 in AGS KD cells. AGS KD cells were co-transfected with either pcDNA p65 or pcDNA 3.1 plus IgκB-Luc and phRL-null as internal control. Cells were harvested 48 h post transfection and NF-κB luciferase activity, normalised for Renilla luciferase activity, was expressed relative to control transfected cells (***, P<0.001). Bottom panel shows immunoblot analysis of p65 and actin 48 h post transfection. (1.72 MB TIF) [file pone.0012038.s004.tif]
